# A novel method to predict visual field progression more accurately, using intraocular pressure measurements in glaucoma patients

**DOI:** 10.1038/srep31728

**Published:** 2016-08-26

**Authors:** The Japanese Archive of Multicentral Database in Glaucoma (JAMDIG) construction group, Ryo Asaoka, Yuri Fujino, Hiroshi Murata, Atsuya Miki, Masaki Tanito, Shiro Mizoue, Kazuhiko Mori, Katsuyoshi Suzuki, Takehiro Yamashita, Kenji Kashiwagi, Nobuyuki Shoji

**Affiliations:** 1 Department of Ophthalmology, The University of Tokyo, Tokyo Japan.; 2 Department of Ophthalmology, Osaka University Graduate School of Medicine, Osaka, Japan.; 3 Department of Ophthalmology, Shimane University Faculty of Medicine, Shimane, Japan.; 4 Division of Ophthalmology, Matsue Red Cross Hospital, Shimane, Japan.; 5 Department of Ophthalmology, Ehime University Graduate School of Medicine, Ehime, Japan.; 6 Department of Ophthalmology, Kyoto Prefectural University of Medicine, Kyoto, Japan.; 7 Department of Ophthalmology, Yamaguchi University Graduate School of Medicine, Yamaguchi, Japan.; 8 Department of Ophthalmology, Kagoshima University Graduate School of Medical and Dental Sciences, Kagoshima, Japan.; 9 Department of Ophthalmology, University of Yamanashi Faculty of Medicine, Yamanashi, Japan.; 10 Orthoptics and Visual Science, Department of Rehabilitation, School of Allied Health Sciences, Kitasato University, Kanagawa, Japan.

## Abstract

Visual field (VF) data were retrospectively obtained from 491 eyes in 317 patients with open angle glaucoma who had undergone ten VF tests (Humphrey Field Analyzer, 24-2, SITA standard). First, mean of total deviation values (mTD) in the tenth VF was predicted using standard linear regression of the first five VFs (VF_1-5_) through to using all nine preceding VFs (VF_1-9_). Then an ‘intraocular pressure (IOP)-integrated VF trend analysis’ was carried out by simply using time multiplied by IOP as the independent term in the linear regression model. Prediction errors (absolute prediction error or root mean squared error: RMSE) for predicting mTD and also point wise TD values of the tenth VF were obtained from both approaches. The mTD absolute prediction errors associated with the IOP-integrated VF trend analysis were significantly smaller than those from the standard trend analysis when VF_1-6_ through to VF_1-8_ were used (p < 0.05). The point wise RMSEs from the IOP-integrated trend analysis were significantly smaller than those from the standard trend analysis when VF_1-5_ through to VF_1-9_ were used (p < 0.05). This was especially the case when IOP was measured more frequently. Thus a significantly more accurate prediction of VF progression is possible using a simple trend analysis that incorporates IOP measurements.

Glaucoma is one of the leading causes of loss of sight in the world^1^,^2^. Glaucoma is a progressive and irreversible optic neuropathy which can result in irreversible visual field (VF) damage and it is therefore essential to accurately predict future VF progression when making glaucoma treatment decisions. Future VF deterioration is usually predicted by applying ordinary least squares linear regression (OLSLR) to global VF indices such as mean deviation (MD), as in the Guided Progression Analysis™ (GPA) software on the Humphrey Field Analyzer (HFA, Carl Zeiss Meditec AG, Dublin, CA, USA). We have reported several alternative approaches to improve the prediction accuracy of VF progression, such as robust regression^3^, least absolute shrinkage and selection operator (Lasso) regression^4^, cluster-wise regression^5^,^6^, and also variational Bayesian linear regression^7^. In addition, many other successful efforts have been reported from other researchers^8^,^9^,^10^,^11^,^12^,^13^,^14^, nonetheless ordinary OLSLR is still very frequently used at the clinical setting. One thing that all these approaches have in common is that VF progression is predicted using only VF measurements; however, other parameters are routinely captured in the clinic which may be useful to supplement any model of VF progression. In particular, previous studies strongly suggest that intraocular pressure (IOP) is closely related to VF deterioration^15^,^16^,^17^,^18^,^19^ and indeed, glaucoma management is usually focused on adequately controlling IOP, nonetheless there is no method to predict VF progression in which both of VF and IOP records are concurrently considered. In the current study, a novel VF trend analysis is carried out by integrating time (the standard independent variable in VF trend analyses) with IOP measurements; we simply multiple time by IOP when this measurement is available. We term this approach ‘IOP-integrated VF trend analysis’ and here we compare the prediction accuracy of the method against the standard VF trend analysis.

## Method

The review board of the University of Tokyo, Osaka University Graduate School of Medicine, Shimane University Faculty of Medicine, Matsue Red Cross Hospital, Ehime University Graduate School of Medicine, Kyoto Prefectural University of Medicine, Yamaguchi University Graduate School of Medicine, Kagoshima University Graduate School of Medical and Dental Sciences, and University of Yamanashi Faculty of Medicine, Kitasato University reviewed and approved all protocols. The study complied with the tenets of the Declaration of Helsinki. Written consent was given by patients for their information to be stored in the hospital database and used for research or shown posted at the outpatient clinic to notify the participants of the study, based on the regulations of the Japanese Guidelines for Epidemiologic Study 2008 issued by the Japanese Government.

### Subjects

#### Subjects and visual fields

VF data were retrospectively obtained from a total of 481 eyes in 309 patients with open angle glaucoma and ten eyes from eight patients with exfoliation glaucoma. All of the data in the current study were collected in the JAMDIG study (Japanese Archive of Multicentral Databases in Glaucoma)^4^ in which nine institutes in Japan were involved. Each eye was VF tested at least 10 times, excluding the first VF (for learning effects). Diagnosis of glaucoma was determined when the following findings were present: 1) presence of typical glaucomatous changes in the optic nerve head, such as a rim notch with a rim width ≤0.1 disc diameters or a vertical cup-to-disc ratio of >0.7 and/or a retinal nerve fiber layer defect with its edge at the optic nerve head margin greater than a major retinal vessel, diverging in an arcuate or wedge shape. VF measurements were performed using the HFA with either the 30-2 or 24-2 program and the Swedish Interactive Threshold Algorithm Standard, but only the 52 test locations overlapping with the 24-2 test pattern were used in the analyses when VFs were obtained with the 30-2 test pattern. Other inclusion criteria were best corrected visual acuity better than 6/12, refraction within ±6 diopter ametropia, no previous ocular surgery except for cataract extraction and intraocular lens implantation, and no other anterior and posterior segment of the eye disease that could affect the VF, including cataract other than clinically insignificant senile cataract. Reliability criteria for VFs were applied: fixation losses less than 20% and false-positive responses less than 15% following the criteria used by the HFA software; false negatives (FN) were not used in as an exclusion criterion^20^. The VF of a left eye was mirror-imaged to that of a right eye for statistical analyses. Eyes that did not have at least seven IOP measurements were removed from the analysis.

### Statistical analysis

#### Standard VF trend analysis

First, a conventional trend analysis was conducted on each series. Linear regressions were carried out using measurements in the first five VFs (VF_1-5_) to predict the corresponding measurement in the tenth VF (VF_10_) for: (i) point-wise total deviation (TD) values and (ii) mean of 52 TD values correspond to HFA 24-2 VF (mTD). Then the difference between the predicted value and the actual value in VF_10_ was calculated. The same procedure was iterated using different lengths of VF series for the predictions: VF_1-6_, VF_1-7_, VF_1-8_ and VF_1-9_. Prediction accuracy was compared using the absolute prediction error for mTD value and root mean squared error (RMSE) for point wise TD value:


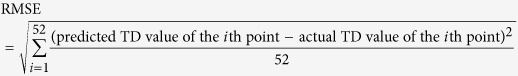


#### IOP-integrated VF trend analysis

**Figure 1:**
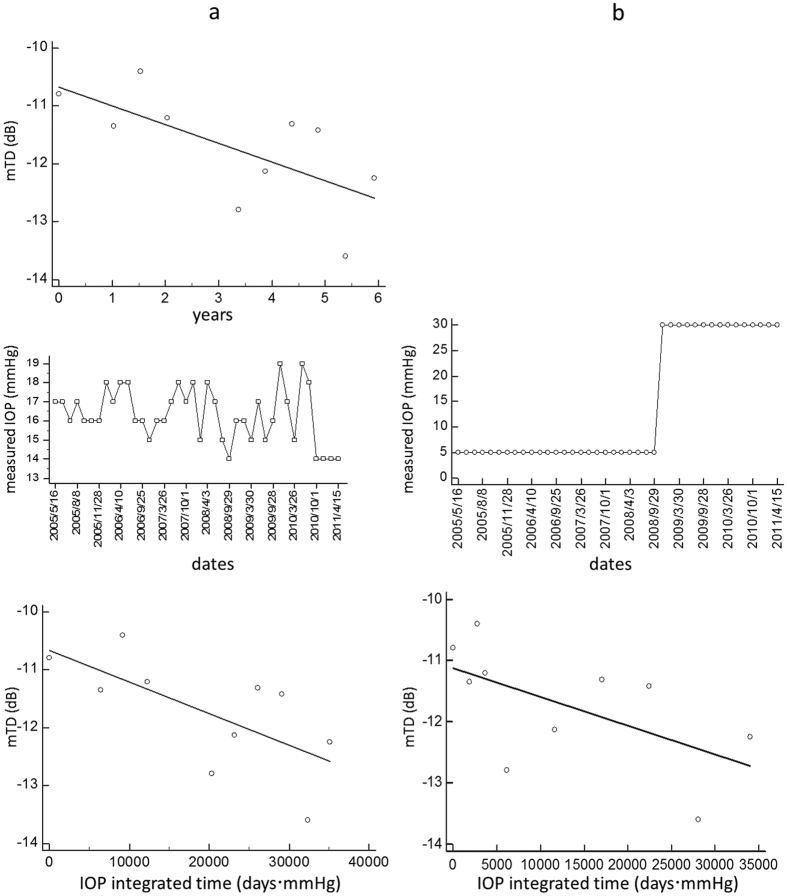
Calculation of IOP-integrated time. IOP-integrated time was calculated by integrating IOP. (**a**) A case example. The top figure shows the time trend analysis in which VF measurements were regressed against time (year). The middle line graph shows the measured IOP values and the bottom figure shows the time integrated time trend analysis. (**b**) Simulated example assuming measured IOP values were 5 mmHg until 2008/9/29 and 30 mmHg after that date. The top figure shows the the line graph of the simulated IOP values and the middle figure shows time integrated time trend analysis. IOP: intraocular pressure, VF: visual field.

**Figure 2:**
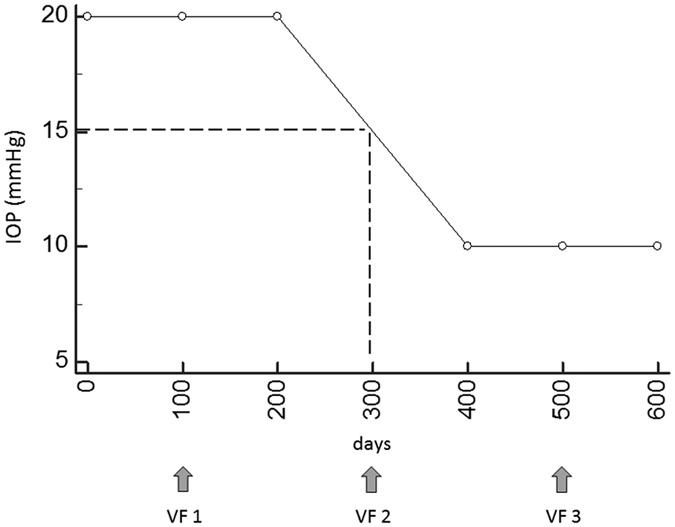
Imputation of missing IOP value on the VF measurement day. In this case, VF was measured on days 0, 300 and 500. An IOP measurement was missing on day 300 so the value was imputed using linear interpolation, IOP: intraocular pressure, VF: visual field.

As shown in Fig. 1, the time from the baseline VF was integrated using IOP measurements. This is a simple calculation of the time multiplied by the IOP value. Figure 1a shows a case example. The top figure shows the time trend analysis in which VF measurements were regressed against time (year). The middle line graph shows the measured IOP values while the bottom figure shows the time integrated time trend analysis in which VF measurements were regressed against time IOP (days⋅mmHg). Figure 1b shows a simulated case for the same case example, assuming measured IOP values were 5 mmHg until 2008/9/29 and 30 mmHg after that date. The top figure shows the IOP integrated time trend analysis and the middle figure is a line graph of the simulated IOP values. If an IOP measurement was not carried out on the date of VF measurement, as is often experienced in the real world clinic, then an IOP for that date was calculated using a linear interpolation of IOP values at the last and next IOP measurement (see Fig. 2). If there were more multiple IOP records between VF measurements, the IOP-integrated time value was calculated for each IOP interval. IOP was always measured with Goldmann tonometry. Linear regression was then performed just like a conventional VF trend analysis; only the IOP-integrated time value was used as the independent variable, instead of time. RMSE and absolute prediction errors associated with point wise and mTD regressions were then calculated as before using VF_1-5_, VF_1-6_, VF_1-7_, VF_1-8_ and VF_1-9_.

The absolute prediction errors for each trend method were compared using linear mixed modelling, whereby patients were treated as a ‘random effect’. Furthermore, the relationship between (i) the IOP measurement interval and (ii) mean IOP, and prediction errors was also investigated using the linear mixed model.

All analyses were performed using the statistical programming language ‘R’ (R version 3.1.3; The Foundation for Statistical Computing, Vienna, Austria).

## Result

Characteristics of the study population are summarized in Table 1. The average mTD at baseline was −6.7 ± 6.2 dB (mean ± standard deviation) and the mean initial patient age was 54.6 ± 12.0 years. The average progression rate of mTD was −0.16 ± 0.42 dB/year. IOP was measured 24.0 ± 8.5 times in 5.5 ± 1.2 years. The mean ± standard deviation of IOP measurements was 13.6 ± 2.2 mmHg.

**Figure 3:**
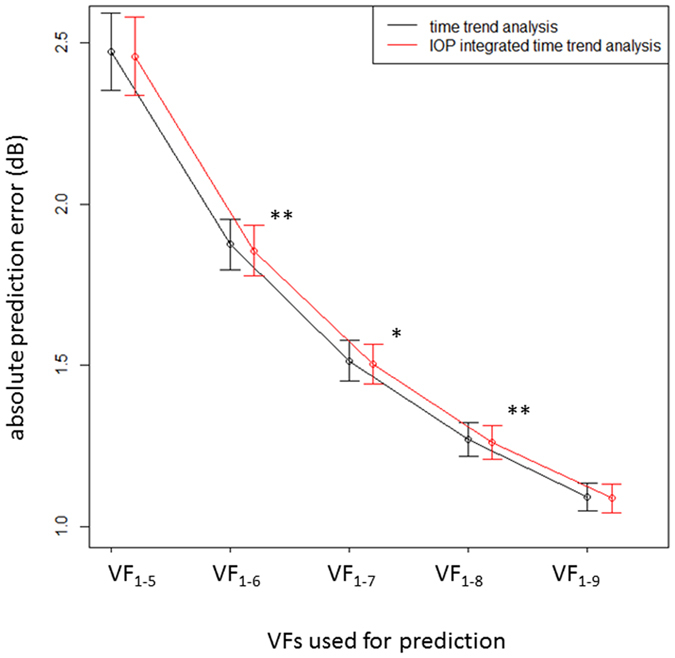
The absolute mTD prediction errors associated with a standard trend analysis and the IOP-integrated VF trend analysis. *p < 0.05, **p < 0.01, IOP: intraocular pressure, mTD: mean of 52 total deviation values correspond to 24-2 Humphrey visual field.

Figure 3 shows the absolute mTD prediction errors associated with the conventional trend analysis as well as the IOP-integrated VF trend analysis. The absolute prediction errors were significantly smaller for the latter approach when VF_1-6_ through to VF_1-8_ were used in the prediction (P = 0.0037, 0.0410, 0.0062, linear mixed model).

**Figure 4:**
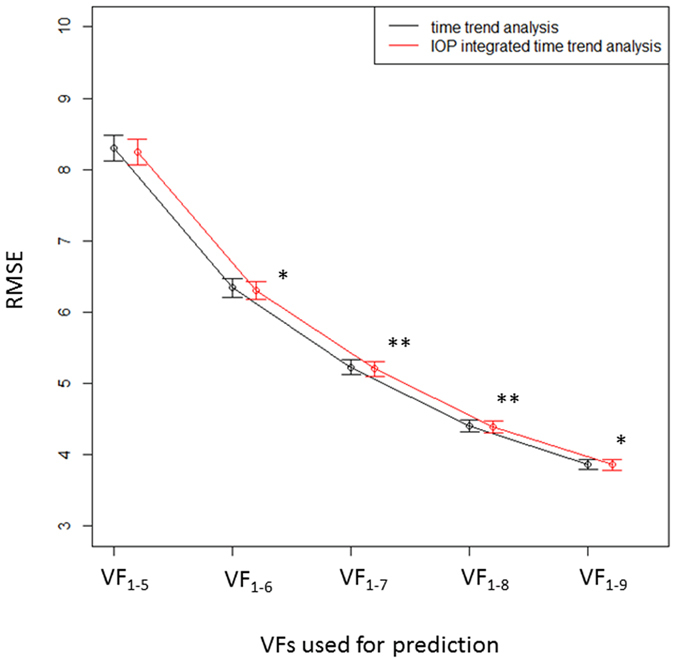
The RMSEs associated with the standard trend analysis and the IOP-integrated VF trend analysis using point-wise mTD values. *p < 0.05, **p < 0.01. OLSLR: ordinal least square regression, IOP: intraocular pressure, PW: point wise, mTD: mean of 52 total deviation values correspond to 24-2 Humphrey visual field.

Figure 4 shows the RMSEs associated with both trend methods when predicting point-wise TD values. The RMSEs with the IOP-integrated VF trend analysis were significantly smaller than those from the standard analysis when VF_1-6_ through to VF_1-9_ were used for prediction (P = 0.0022, 0.0216, 0.0057, 0.048, linear mixed model).

Table 2 summarizes the difference between the absolute mTD prediction errors obtained with both trend analyses, and the relationship between this difference and the IOP measurement interval and mean IOP. As can be seen in Table 2, there was a significant relationship between the magnitude of the difference and IOP measurement interval for VF_1-6_ and VF_1-7_ (p = 0.014 and 0.0077, respectively, linear mixed model). The relationship approached significance (p value of 0.0503) for VF_1-5_. No significant relationship was observed between the magnitude of the difference in errors and mean IOP (p > 0.05, linear mixed model).

## Discussion

In this study, a novel VF trend analysis was proposed that considers IOP measurements in the regression. A significantly smaller prediction error was observed for this approach compared to a standard time trend analysis. Furthermore, the method appears not to be significantly affected by the level of IOP or progression rate. However, despite a significant reduction in prediction error, the magnitude of this improvement is small relative to previous novel regression models that use only VF data^3^,^4^,^5^,^6^,^7^,^8^,^9^,^10^. One of the possible reasons for such a small improvement is that IOP fluctuates in the short and long term^21^ and measuring IOP at the clinic in approximately three month intervals is too infrequent to observe the true trend in IOP. In addition, the magnitude of the interval between VF tests may be directly related to the previous IOP measurement (a person with high IOP is likely to be seen again sooner), and hence the influence of IOP is already reflected to some extent in the standard trend analysis.

It should be noted that the VF data analyzed in this study was obtained from real world clinics. The mean VF progression rate in the current study was −0.16 dB/year with a mean IOP of 13.6 mmHg, suggesting IOP was, in general, well-managed. A further study should be carried out using a dataset with a faster average progression rate and also a higher average IOP.

It has been suggested that glaucomatous optic disc change occurs as a result of the difference between IOP and cerebrospinal pressure^22^, although this hypothesis remains controversial^23^. We carried out a further trend analysis using IOP subtracted cerebrospinal pressure (the IOP value was subtracted from the average cerebrospinal pressure in a normal population, calculated as the mean of the upper and lower limits for normal range cerebrospinal pressure), however, no further improvement of prediction error was observed (data not shown). In addition, corneal thickness and corneal hysteresis are closely related to Goldmann tonometry measurements^24^,^25^,^26^,^27^,^28^ and also VF progression^29^,^30^. A future study should be carried out using IOP measurements that have been adjusted for these factors. A previous study reported that incorporating other risk factors, namely mean IOP, central corneal thickness, and presence of progressive optic disc damage, resulted in a marked improvement in the prediction of VF progression^31^. The improvement in the current study was much smaller than this previous report, however, the virtue of the approach outlined is its simplicity: the standard linear regression model is all that is needed, whereas a Bayesian modelling approach was implemented in the previous study.

We observed that the difference between absolute mTD prediction errors obtained with the standard trend analysis and the IOP-integrated VF trend analysis were larger when the IOP measurement interval was greater. Thus, it appears that measuring IOP more frequently is advantageous when predicting future VF progression. IOP is known to change every day and throughout the day^32^,^33^. It would be interesting to see whether including several IOP measurements throughout the day, such as IOP monitoring for 24 hours^32^,^33^, would improve the results. We noted that absolute mTD prediction errors obtained with standard trend analysis were larger than those from the IOP-integrated method when the progression rate was fast (data not shown). This is likely because VF progression is related to IOP level as suggested by numerous previous studies^34^. Various methods have been proposed to measure VF progression in glaucoma patients^3^,^4^,^7^,^9^,^11^,^12^,^13^,^14^,^35^,^36^, however, simple time trend analysis remains the standard in clinical settings. Hence we compared the prediction performance of the IOP integrated trend analysis against the standard approach. It should be further investigated whether combining the current approach with other novel regression models further improves prediction accuracy. In addition, linear interpolation was used to calculate the missing IOP using the IOP values at the last and next IOP measurement (see Fig. 2). Other methods could give more accurate estimation, such as spline interpolation, and the effect of the interpolation methods on the prediction accuracy of the VF sensitivity should be carried out in a future study, although this effect may be small, having the stable IOP control (13.6 ± 2.2 mmHg) under the treatment.

In conclusion, we have demonstrated a simple method to incorporate IOP control in VF trend analysis. As a result, a statistically significant improvement in prediction was observed.

## Additional Information

**How to cite this article**: The Japanese Archive of Multicentral Database in Glaucoma (JAMDIG) construction group. A novel method to predict visual field progression more accurately, using intraocular pressure measurements in glaucoma patients. *Sci. Rep.* **6**, 31728; doi: 10.1038/srep31728 (2016).

## Figures and Tables

**Table 1:** Subject demographics.

Demographics	Value
age, mean ± SD (range), years old	54.6 ± 12.0(21 to 82)
sex (male:female), patients	154:163
Right:left, eyes	241:250
Follow-up (year, mean ± SD) (range), year	5.5 ± 1.2(2.1 to 9.4)
IOP measurements carried times (range), times	24.0 ± 8.5(7 to 52)
mTD in the initial VF, dB, mean ± SD (range)	−6.7 ± 6.2(−26.7 to 2.8)
mTD progression rate, dB/year, mean ± SD (range)	−0.16 ± 0.42(−1.8 to 2.2)
Mean of IOP, mmHg, mean ± SD (range)	13.6 ± 2.2(7.7 to 22.1)
SD of IOP, mean ± SD (range)	1.6 ± 0.6(0.7 to 7.7)

mTD: mean of 52 total deviation values correspond to 24-2 Humphrey visual field, SD: standard deviation, IOP: intraocular pressure.

**Table 2:** Difference in absolute mTD prediction errors from each approach, and the relationship between the magnitude of the difference and IOP measurement interval and mean IOP.

VFs used for prediction	VF_1-5_	VF_1-6_	VF_1-7_	VF_1-8_	VF_1-9_
Mean ± SD difference in absolute mTD error (dB)*	0.014 ± 0.23	0.020 ± 0.15	0.010 ± 0.11	0.0095 ± 0.077	0.0037 ± 0.055
coefficient against IOP measurement interval	8.6 × 10^−5^	2.0 × 10^−10^	5.2 × 10^−14^	2.5 × 10^−14^	8.9 × 10^−5^
p value	0.0503	0.014	0.0077	0.22	0.43
coefficient against mean IOP	0.30	0.45	0.54	0.45	−0.0025
p value	0.22	0.30	0.45	0.54	0.45

mTD: mean of 52 total deviation values correspond to 24-2 Humphrey visual field, SD: standard deviation, IOP: intraocular pressure, coefficient and p values were obtained using linear mixed model.

*(absolute mTD error from standard trend analysis) − (absolute mTD error from IOP-integrated trend analysis).

## References

[b1] Quigley H. A. Number of people with glaucoma worldwide. The British journal of ophthalmology 80, 389–393 (1996). 8695555 10.1136/bjo.80.5.389 PMC505485

[b2] Congdon N. *et al.* Causes and prevalence of visual impairment among adults in the United States. Archives of ophthalmology 122, 477–485, doi: 10.1001/archopht.122.4.477 (2004). 15078664

[b3] Taketani Y., Murata H., Fujino Y., Mayama C. & Asaoka R. How Many Visual Fields Are Required to Precisely Predict Future Test Results in Glaucoma Patients When Using Different Trend Analyses? Invest Ophthalmol Vis Sci 56, 4076–4082, doi: 10.1167/iovs.14-16341 (2015). 26114484

[b4] Fujino Y., Asaoka R., Murata H., Miki A. Tanito M., Mizoue S., Mori K., Suzuki K., Yamashita T., Kashiwagi K. & Shoji N. Evaluation of Glaucoma Progression in Large-Scale Clinical Data: The Japanese Archive of Multicentral Databases in Glaucoma (JAMDIG) Construction Group. Invest Ophthalmol Vis Sci. 57(4), 2012–2020, Apr 1, 2016. 27127924 10.1167/iovs.15-19046

[b5] Hirasawa K., Murata H., Hirasawa H., Mayama C. & Asaoka R. Clustering Visual Field Test Points Based on Rates of Progression to Improve the Prediction of Future Damage. Invest Ophthalmol Vis Sci 55, 7681–7685, doi: 10.1167/iovs.14-15040 (2014). 25342611

[b6] Hirasawa K., Murata H. & Asaoka R. Revalidating the Usefulness of a “Sector-Wise Regression” Approach to Predict Glaucomatous Visual Function Progression. Invest Ophthalmol Vis Sci 56, 4332–4335, doi: 10.1167/iovs.15-16694 (2015). 26176870

[b7] Murata H., Araie M. & Asaoka R. A new approach to measure visual field progression in glaucoma patients using variational bayes linear regression. Invest Ophthalmol Vis Sci 55, 8386–8392, doi: 10.1167/iovs.14-14625 (2014). 25414192

[b8] Zhu H. *et al.* Detecting changes in retinal function: Analysis with Non-Stationary Weibull Error Regression and Spatial enhancement (ANSWERS). PloS one 9, e85654, doi: 10.1371/journal.pone.0085654 (2014). 24465636 PMC3894992

[b9] Azarbod P. *et al.* Validation of point-wise exponential regression to measure the decay rates of glaucomatous visual fields. Invest Ophthalmol Vis Sci 53, 5403–5409, doi: 10.1167/iovs.12-9930 (2012). 22743320

[b10] Chen A. *et al.* Models of glaucomatous visual field loss. Invest Ophthalmol Vis Sci 55, 7881–7887, doi: 10.1167/iovs.14-15435 (2014). 25377224

[b11] Liang Z., Tomioka R., Murata H., Asaoka R. & Yamanishi K. Quantitative prediction of glaucomatous visual field loss from few measurements. IEEE 13th International Conference on Data Mining (ICDM) 1121–1126 (2013).

[b12] Maya S., Morino K. & Yamanishi K. Predicting glaucoma progression using multi-task learning with heterogeneous features. IEEE International Conference on Big Data 2014 (IEEE BigData 2014) 261–270 (2014).

[b13] Maya S., Morino K., Murata H., Asaoka R. & Yamanishi K. Discovery of Glaucoma Progressive Patterns Using Hierarchical MDL-Based Clustering. the 21th ACM SIGKDD International Conference on Knowledge Discovery and Data Mining (KDD2015) 1979–1988 (2015).

[b14] Tomoda K., Morino K., Murata H., Asaoka R. & Yamanishi K. Predicting Glaucomatous Progression with Piecewise Regression Model from Heterogeneous Medical Data. HEALTHINF 2016, in press (2016).

[b15] Leske M. C., Hyman L., Hussein M., Heijl A. & Bengtsson B. Comparison of glaucomatous progression between untreated patients with normal-tension glaucoma and patients with therapeutically reduced intraocular pressures. The effectiveness of intraocular pressure reduction in the treatment of normal-tension glaucoma. American journal of ophthalmology 127, 625–626 (1999). 10334369

[b16] Heijl A. *et al.* Reduction of intraocular pressure and glaucoma progression: results from the Early Manifest Glaucoma Trial. Archives of ophthalmology 120, 1268–1279 (2002). 12365904 10.1001/archopht.120.10.1268

[b17] Kass M. A. *et al.* The Ocular Hypertension Treatment Study: a randomized trial determines that topical ocular hypotensive medication delays or prevents the onset of primary open-angle glaucoma. Archives of ophthalmology 120, 701–713, discussion 829-730 (2002). 12049574 10.1001/archopht.120.6.701

[b18] Garway-Heath D. F. *et al.* Latanoprost for open-angle glaucoma (UKGTS): a randomised, multicentre, placebo-controlled trial. Lancet 385, 1295–1304, doi: 10.1016/S0140-6736(14)62111-5 (2015). 25533656

[b19] The Advanced Glaucoma Intervention Study (AGIS): 7. The relationship between control of intraocular pressure and visual field deterioration.The AGIS Investigators. American journal of ophthalmology 130, 429–440 (2000). 11024415 10.1016/s0002-9394(00)00538-9

[b20] Bengtsson B. & Heijl A. False-negative responses in glaucoma perimetry: indicators of patient performance or test reliability? American journal of ophthalmology 130, 689 (2000). 11078863 10.1016/s0002-9394(00)00758-3

[b21] Fogagnolo P. *et al.* Short- and long-term phasing of intraocular pressure in stable and progressive glaucoma. Ophthalmologica 230, 87–92, doi: 10.1159/000351647 (2013). 23796507

[b22] Wang N. *et al.* Orbital cerebrospinal fluid space in glaucoma: the Beijing intracranial and intraocular pressure (iCOP) study. Ophthalmology 119, 2065–2073 e2061, doi: 10.1016/j.ophtha.2012.03.054 (2012). 22749084

[b23] Jonas J. B., Wang N., Yang D., Ritch R. & Panda-Jonas S. Facts and myths of cerebrospinal fluid pressure for the physiology of the eye. Progress in retinal and eye research 46, 67–83, doi: 10.1016/j.preteyeres.2015.01.002 (2015). 25619727

[b24] Ehlers N., Bramsen T. & Sperling S. Applanation tonometry and central corneal thickness. Acta ophthalmologica 53, 34–43 (1975). 1172910 10.1111/j.1755-3768.1975.tb01135.x

[b25] Whitacre M. M. & Stein R. Sources of error with use of Goldmann-type tonometers. Survey of ophthalmology 38, 1–30 (1993). 8235993 10.1016/0039-6257(93)90053-a

[b26] Whitacre M. M., Stein R. A. & Hassanein K. The effect of corneal thickness on applanation tonometry. American journal of ophthalmology 115, 592–596 (1993). 8488910 10.1016/s0002-9394(14)71455-2

[b27] Bron A. M., Creuzot-Garcher C., Goudeau-Boutillon S. & d’Athis P. Falsely elevated intraocular pressure due to increased central corneal thickness. Graefe’s archive for clinical and experimental ophthalmology=Albrecht von Graefes Archiv fur klinische und experimentelle Ophthalmologie 237, 220–224 (1999). 10.1007/s004170050222 10090585

[b28] Brandt J. D., Beiser J. A., Kass M. A. & Gordon M. O. Central corneal thickness in the Ocular Hypertension Treatment Study (OHTS). Ophthalmology 108, 1779–1788 (2001). 11581049 10.1016/s0161-6420(01)00760-6

[b29] Jonas J. B. *et al.* Central corneal thickness correlated with glaucoma damage and rate of progression. Invest Ophthalmol Vis Sci 46, 1269–1274, doi: 10.1167/iovs.04-0265 (2005). 15790889

[b30] Medeiros F. A. *et al.* Corneal thickness as a risk factor for visual field loss in patients with preperimetric glaucomatous optic neuropathy. American journal of ophthalmology 136, 805–813 (2003). 14597030 10.1016/s0002-9394(03)00484-7

[b31] Medeiros F. A. *et al.* Incorporating risk factors to improve the assessment of rates of glaucomatous progression. Invest Ophthalmol Vis Sci 53, 2199–2207, doi: 10.1167/iovs.11-8639 (2012). 22410555 PMC3808189

[b32] Wilensky J. T. The role of diurnal pressure measurements in the management of open angle glaucoma. Curr Opin Ophthalmol 15, 90–92 (2004). 15021217 10.1097/00055735-200404000-00005

[b33] Hughes E., Spry P. & Diamond J. 24-hour monitoring of intraocular pressure in glaucoma management: a retrospective review. J Glaucoma 12, 232–236 (2003). 12782841 10.1097/00061198-200306000-00009

[b34] The effectiveness of intraocular pressure reduction in the treatment of normal-tension glaucoma. Collaborative Normal-Tension Glaucoma Study Group. American journal of ophthalmology 126, 498–505 (1998). 9780094 10.1016/s0002-9394(98)00272-4

[b35] Zhu H., Crabb D. P., Ho T. & Garway-Heath D. F. More Accurate Modeling of Visual Field Progression in Glaucoma: ANSWERS. Invest Ophthalmol Vis Sci 56, 6077–6083, doi: 10.1167/iovs.15-16957 (2015). 26393667

[b36] Russell R. A. & Crabb D. P. On alternative methods for measuring visual field decay: Tobit linear regression. Invest Ophthalmol Vis Sci 52, 9539–9540, doi: 10.1167/iovs.11-8948 (2011). 22180638

